# Catechol-O-Methyltransferase moderates effect of stress mindset on affect and cognition

**DOI:** 10.1371/journal.pone.0195883

**Published:** 2018-04-20

**Authors:** Alia J. Crum, Modupe Akinola, Bradley P. Turnwald, Ted J. Kaptchuk, Kathryn T. Hall

**Affiliations:** 1 Department of Psychology, Stanford University, Stanford, CA, United States of America; 2 Columbia Business School, Columbia University, New York, NY, United States of America; 3 Division of General Medicine and Primary Care, Beth Israel Deaconess Medical Center, Harvard Medical School, Boston, MA, United States of America; 4 Program in Placebo Studies, Beth Israel Deaconess Medical Center, Harvard Medical School, Boston, MA, United States of America; 5 Division of Preventive Medicine, Brigham and Women's Hospital, Harvard Medical School, Boston, MA, United States of America; University of Texas Health Science Center at Houston, UNITED STATES

## Abstract

There is evidence that altering stress mindset—the belief that stress is enhancing vs. debilitating—can change cognitive, affective and physiological responses to stress. However individual differences in responsiveness to stress mindset manipulations have not been explored. Given the previously established role of catecholamines in both placebo effects and stress, we hypothesized that genetic variation in catechol-O-methyltransferase (COMT), an enzyme that metabolizes catecholamines, would moderate responses to an intervention intended to alter participants’ mindsets about stress. Participants (N = 107) were exposed to a stress mindset manipulation (videos highlighting either the enhancing or debilitating effects of stress) prior to engaging in a Trier Social Stress task and subsequent cognitive tasks. The associations of the *COMT* rs4680 polymorphism with the effect of stress mindset video manipulations on cognitive and affective responses were examined. Genetic variation at rs4680 modified the effects of stress mindset on affective and cognitive responses to stress. Individuals homozygous for rs4680 low-activity allele (met/met) were responsive to the stress-is-enhancing mindset manipulation as indicated by greater increases in positive affect, improved cognitive functioning, and happiness bias in response to stress. Conversely, individuals homozygous for the high-activity allele (val/val) were not as responsive to the stress mindset manipulation. These results suggest that responses to stress mindset intervention may vary with *COMT* genotype. These findings contribute to the understanding of gene by environment interactions for mindset interventions and stress reactivity and therefore warrant further investigations.

## Introduction

Cognitive, emotional, and physiological responses to stress are not solely determined by the amount of stress one experiences but also by one’s beliefs about stress. Research on stress mindset—the belief that stress has enhancing versus debilitating properties—has demonstrated that higher indices of health, performance, and well-being can ensue from holding a stress-is-enhancing relative to a stress-is-debilitating mindset [1]. SIE and SID mindsets can also differentially affect physiological and behavioral responses under stress, with SIE mindsets engendering more adaptive responses (e.g., reduced cortisol reactivity) and more approach-related behavior (e.g., greater desire for feedback from both peers and experts on their “charisma” during a public speaking task) [1]. Importantly, evidence suggests that stress mindset can be changed to improve stress responses. Prior research has demonstrated that participants adopting a SIE mindset, after merely watching a 3-minute video highlighting enhancing (vs. debilitating) effects of stress, demonstrated greater cognitive flexibility, heightened positive affect, and increased anabolic hormonal reactivity in response to an acute stressor [2] relative to those adopting a SID mindset. Further, watching three short (3-minute) videos emphasizing the beneficial aspects of stress at work was associated with adopting a SIE mindset as well as improvements in work performance and self-reported health [1].

Although research on stress mindset is growing rapidly, the source of variability in individual responses remains unexplored. Identification of genetic polymorphisms associated with sensitivity to stress mindset offers one approach to identifying subsets of the population that can be differentially influenced by stress mindset manipulations. A rich literature connecting catecholamine function to stress implicates the catecholamine regulatory system as a strong candidate for moderating responses to stress mindset interventions. Stress induces adreno-medullary catecholamine secretion [3] and affects catecholamine signaling in the prefrontal cortex (PFC), where conditions of acute stress impair PFC operations via excessive dopamine and norepinephrine release [4–9]. Catechol-O-methyltransferase (COMT) is an enzyme that metabolizes catecholamines including dopamine, epinephrine and norepinephrine. The most well-studied polymorphism in *COMT* is rs4680, which encodes either a G (valine or val) high-activity or A (methionine or met) low-activity form of the enzyme [10]. Early work implicated variation at rs4680 in deficits in cognitive functioning and emotional processing characteristic of patients with a variety of mental disorders (e.g., schizophrenia, bipolar disorder) compared to healthy controls. However, mounting evidence suggests that variation in *COMT* is more likely associated with these specific cognitive and affective endophenotypes rather than with the complex diseases themselves [11].

Neuromodulation of stress reactivity, coping and placebo responses are mediated in large part by catecholamines in the brain and at target organs like the heart. In the brain, the prefrontal cortex is important for higher-order cognitive functions engaged in appraisal of environmental stressors and is also sensitive to the detrimental effects of stress [12]. COMT is the primary dopamine metabolic enzyme in the prefrontal cortex. Hence variation in dopamine metabolism caused by functional variation in COMT can result in individual differences in cognitive functions as well as stress responses. Several studies on stress and COMT have reported that whereas met-allele homozygosity is associated with higher levels of psychosocial stress [13, 14] or stress from early life adversity [15, 16], the effect among val allele homozyogotes is blunted. Met allele homozygosity is also associated with greater anxiety, reactivity to emotional faces [17, 18], and pain sensitivity [15].

In summary, a growing field of research, including differential susceptibility [19] and placebo response [20] have implicated genetic variation at *COMT* rs4680 as a broader potential neurogenetic link between social and interpersonal environmental cues and cognitive, emotional, and physiological responses. In line with the differential susceptibility framework [21], stress mindset theory explores whether certain individuals have a higher likelihood of being negatively affected by adverse conditions (i.e., stress) and could disproportionately benefit from constructive interventions or environments (i.e., SIE mindset). Recent research has revealed genetic moderation of not only responses to adversity, but also the efficacy of interventions [22]. Here we examine how genetic variation at rs4680 influences the affective and cognitive responses to stress after receiving a SIE or SID mindset manipulation, and examine moderation of rs4680 genotype on the efficacy of the SIE intervention. Given the specific effects of stress on catecholamine release and placebo response, we hypothesize that genetic variation in *COMT* may be a potential moderator of stress mindset effects such that met/met individuals will be more responsive to mindset interventions or manipulations suggesting that stress is enhancing versus debilitating.

## Materials and methods

### Participants

Based on power analysis based on the average effect size (*d* = .66) found in previous stress reappraisal manipulations [1], 124 participants were recruited from a university study pool for a study on “Stress and Performance.” Participants received $20 for their participation. This study explored the subset of 107 participants who consented to be genotyped (Table 1). The Columbia University Institutional Review Board reviewed and approved of all procedures. Informed consent was obtained from all subjects.

**Table 1:** Demographic characteristics (N = 107).

		Genotyped participants	met/met 15 (14%)	val/met 51 (48%)	val/val 41 (38%)
**Demographics**					
**Female N (%)**		70 (65.4)	10 (66.7)	33 (64.7)	27 (65.9)
**Age**		24.1 (5.1)	24.8 (5.6)	23.2 (5.7)	24.6 (4.2)
**Race N (%)**					
**White**		41 (38.3)	6 (40.0)	25 (49.0)	10 (24.4)
**Asian**		32 (29.9)	2 (13.3)	12 (23.5)	18 (43.9)
**Black**		19 (17.8)	1 (6.7)	9 (17.6)	9 (22.0)
**Other**		15 (14.0%)	6 (40.0)	5 (9.8)	4 (9.8)
**Baseline Characteristics**					
**Stress Mindset**	1.81 (.63)		1.54 (.46)	1.88 (.61)	1.81 (.69)
**Affect**					
**Positive**	2.99 (.75)		3.19 (.49)	3.03 (.80)	2.79 (.75)
**Negative**	1.53 (.49)		1.67 (.60)	1.44 (.45)	1.58 (.50)

### Procedure

After arriving at the laboratory, the experimenter reviewed the procedures and risks documented in the consent form with each participant, following which participants were given time to review and sign. Participants were randomized using a random number generator to either a SIE or SID mindset manipulation elicited through a 3-minute multi-media video using words, music, and corresponding images to emphasize either the enhancing or deleterious properties of stress on cognitive performance [1]. All statements in the videos were based on published research but biased toward either the enhancing or debilitating effects of stress. For example the SIE video stated “the stress response pumps adrenaline throughout your body fueling the brain with blood and oxygen, increasing focus and heightening alertness and is designed to enhance your performance”, and included scenarios such as doctors demonstrating skilled performances during stressful surgeries. In contrast, the SID video stated “the stress response pumps adrenaline throughout your body; this response is designed to prepare you for physical action but it can hijack your ability to think clearly and diminish your capacity to solve problems”, and included scenarios such as doctors making grave medical errors under stress and job-related accidents that can occur under stress. Complete videos can be viewed at https://mbl.stanford.edu/instruments/stress-mindset-manipulation-videos. After watching the videos, participants engaged in a modified Trier Social Stress Task (TSST) [23] in which the participant was asked to deliver a speech in a mock job interview (in front of one male and one female interviewer) followed by a question and answer session in which they were randomly assigned to receive either positive or negative feedback. Participants’ mood was assessed at five time points throughout the session (see Outcome Measures below for specific details about the timing of these measures). After participants completed the TSST they engaged in a series of cognitive tasks including measures of attentional bias (dot-probe task described in more detail below) and cognitive interference (Stroop task, described in more detail below). Genotype effects were examined for SIE vs. SID conditions. The current manuscript reports analyses on the moderating role of the *COMT* genotype in shaping affective and cognitive outcomes to the stress mindset manipulation. Because the goal of this manuscript was to explore the moderating role of COMT in determining the effects of mindset and we did not have the power to detect COMT x mindset x feedback condition effects, we collapsed across the positive and negative feedback conditions and controlled for any effects of feedback in all analyses. Additional details on the procedure and results from the main effects of mindset and feedback manipulation are reported elsewhere [2].

### Measures

#### Self-report measures

Stress mindset was assessed at baseline and following the video manipulation using the Stress Mindset Measure [1]. Participants rated agreement with eight statements regarding the effect of stress on a 0–4 Likert scale. Self-reported positive and negative affect were assessed using the Positive and Negative Affect Scale (PANAS) [24] at five time-points: (1) upon arrival (baseline), (2) after watching the stress mindset videos, (3) after receiving speech task instructions, (4) after the speech task, and (5) after the question and answer component of the speech task. Participants rated their feelings on twenty emotional states (ten positive; ten negative) on a 1 (not at all) to 5 (a great deal) scale. “Positive affect” (alphas range from .89 to .92 across time points) and “negative affect” (alphas range from .80 to .85) scales were calculated, including the items suggested by Watson and Clark [25].

#### Cognitive performance measures

To assess visual attention to positive and negative stimuli, participants engaged in a computerized dot-probe task [26]. Black and white pictures of white male faces identical to those used in Bradley et al [27] served as stimuli. Reaction time to the probe was used to assess attentional bias. Exposure to the facial expression of the stimuli (happy, angry, or neutral) and target dot position (right or left of fixation) were randomized across all 80 trials presented and latencies were recorded by computer [1].

Cognitive interference was measured using the Stroop color-naming task [28, 29]. The Stroop task is commonly used to examine one’s ability to inhibit cognitive interference that occurs when people attempt to process the features of one stimulus (i.e. names of a color written in words) while another feature (i.e. the ink color the word is printed in) may be interfering. Participants completed 20 practice and 90 experimental trials and were asked to correctly identify the name of the color written in words. Stroop interference scores were computed as the difference in response latencies (in milliseconds) between incongruent (i.e. the ink color of the word was different than the written word, for example “green” written in yellow ink) and congruent trials (i.e. the ink color of the word was the same as the written word, for example “green” written in green ink), with higher scores indicating greater cognitive interference. On the basis of procedures used in previous studies, incorrect responses and latencies above 2000 ms and below 200 ms were recoded as missing data [28–30].

#### Genotyping

Genomic DNA was extracted from saliva using the Qiagen kit (Valencia, CA) following the manufacturer’s protocol. TaqMan SNP Genotyping assays were purchased from Applied Biosystems, (Foster City, CA), and reads were obtained on rs4680 following the manufacturer’s protocol on an Applied Biosystems 7900HT instrument, using SDS version 2.4 software.

### Statistical analysis

Hardy–Weinberg Equilibrium (HWE) and linkage disequilibrium were calculated using the Online Encyclopedia for Genetic Epidemiology studies [31, 32]. We used a gene dosage model for ‘‘*COMT* genotype”, that coded each participant’s rs4680 genotype as follows: 0 = met/met; 1 = val/met; 2 = val/val. ANOVAs for all dependent variables 2 (mindset: SIE vs. SID) x 3 (*COMT* rs4680 genotype: met/met vs. val/met vs. val/val) were conducted. Where there were multiple assessments (postitive and negative affect), we conducted repeated measures ANOVAs with time as a within subjects variable and mindset and genotype as a between subjects variable. In cases where Mauchly’s test of sphericity was violated, degrees of freedom were corrected using Greenhouse-Geisser estimates of sphericity. To further understand interactions, the sample was stratified by genotype to examine how the mindset manipulation differentially affected met/met vs. met/val vs. val/val participants and then stratified by mindset to understand how the effects of genotype were different in SIE and SID conditions. Where there were multiple comparisons, univariate ANOVAs with Bonferroni corrected post hoc comparisons were used to test differences between genotype for the SIE and SID conditions separately. We controlled for baseline stress mindset and feedback condition (0 = positive 1 = negative) in the stress task in all regression models. Gender (coded as 0 = female, 1 = male) was included as a covariate if it was indicated as a significant predictor of the dependent variable. Gender was a significant predictor for cognitive interference but no other dependent variable. Effects with *p-*values **≤** .05 were considered statistically significant. Effects with *p-*values **≤** .10 were considered marginally significant.

## Results

### Baseline characteristics

The *COMT* rs4680 minor allele (A or met-allele) frequency was 0.38 and the SNP was in Hardy-Weinberg Equilibrium (*p* = 0.75), with the following distribution: 14% met/met, 48% val/met, and 38% val/val. Demographics are described in Table 1. Participants were 65.4% female; mean age = 24.09 years; SD = 5.17 and there were no significant demographic differences across *COMT* rs4680 genotypes.

Baseline stress mindset did not vary by *COMT* genotype (*F*_1,107_ = 1.54, *p* = .22, η^2^ = .030). There were no significant differences by genotype on baseline levels of positive affect (*F*_1,107_ = 1.98, *p* = .14, η^2^ = .037), or negative affect (*F*_1,107_ = 1.79, *p* = .17, η^2^ = .033) as measured by the PANAS (Table 1).

### Mindset manipulation

The stress mindset video manipulation produced significant changes in mindset as expected; participants randomized to SIE reported an increased SIE mindset whereas participants randomized to SID reported an increased SID mindset post-manipulation (*F*_1,96_ = 92.9, *p* < .001, η^2^ = .492). The changes in mindset did not differ by *COMT* rs4680 genotype (*F*_2,96_ = 1.15, *p* = .32, η^2^ = .024).

### Changes in affect

**Fig 1:**
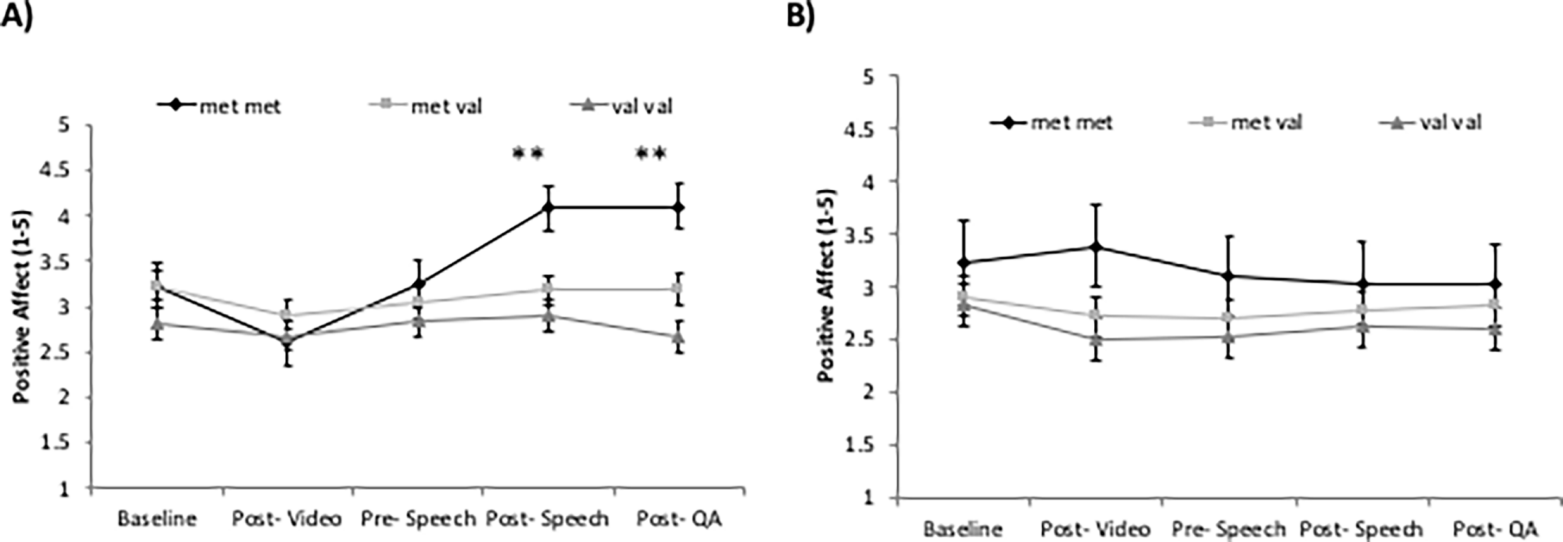
**Effects of genotype on positive affect in SIE (A) and SID (B) conditions.** There is a significant time x genotype effect in the SIE condition (*p* ≤.01) (A) and not in the SID condition (B) Asterisks indicate significant differences at each time point between genotype in both SIE and SID conditions using Bonferroni corrected post hoc comparisons (** *p* **≤**.01; * *p* **≤** .05) revealing that differences in positive affect occurred post-speech and post-Q and A. The time x genotype x mindset effect is significant at *p* **≤** .05. Error bars represent standard errors of the means.

For positive affect, we observed a significant time x genotype x mindset effect (*F*_6.2,283.1_ = 2.35, *p* = .030, η^2^ = .049). Simple effects splitting the sample by genotype revealed a significant time x mindset effect (*F*_4,32_ = 3.52, *p* = .017, η^2^ = .306) for met/met individuals in the SIE condition compared to met/met individuals in the SID condition. In contrast, there were no significant effects of mindset on positive affect for met/val or val/val participants. Simple effects splitting the sample by mindset condition revealed a significant time x genotype effect in the SIE condition (*F*_6.4,133.9_ = 3.06, *p* = .007, η^2^ = .127) (Fig 1A), whereas there was no significant effect of genotype in the SID condition (Fig 1B). Univariate ANOVAs for each time point with Bonferroni corrected post hoc tests examining differences between genotype in both SIE and SID conditions revealed no significant differences between genotype for SIE or SID conditions at time points 1 (baseline), 2 (post stress mindset manipulation), or 3 (pre-speech) (Fig 1A). However, met/met individuals in the SIE condition reported significantly higher positive affect than val/val individuals at time-points 4 (post-speech) (p = .033) and 5 (post-Q & A) (p = .005). We observed no significant time x mindset x genotype for negative affect.

### Cognitive tasks

**Fig 2:**
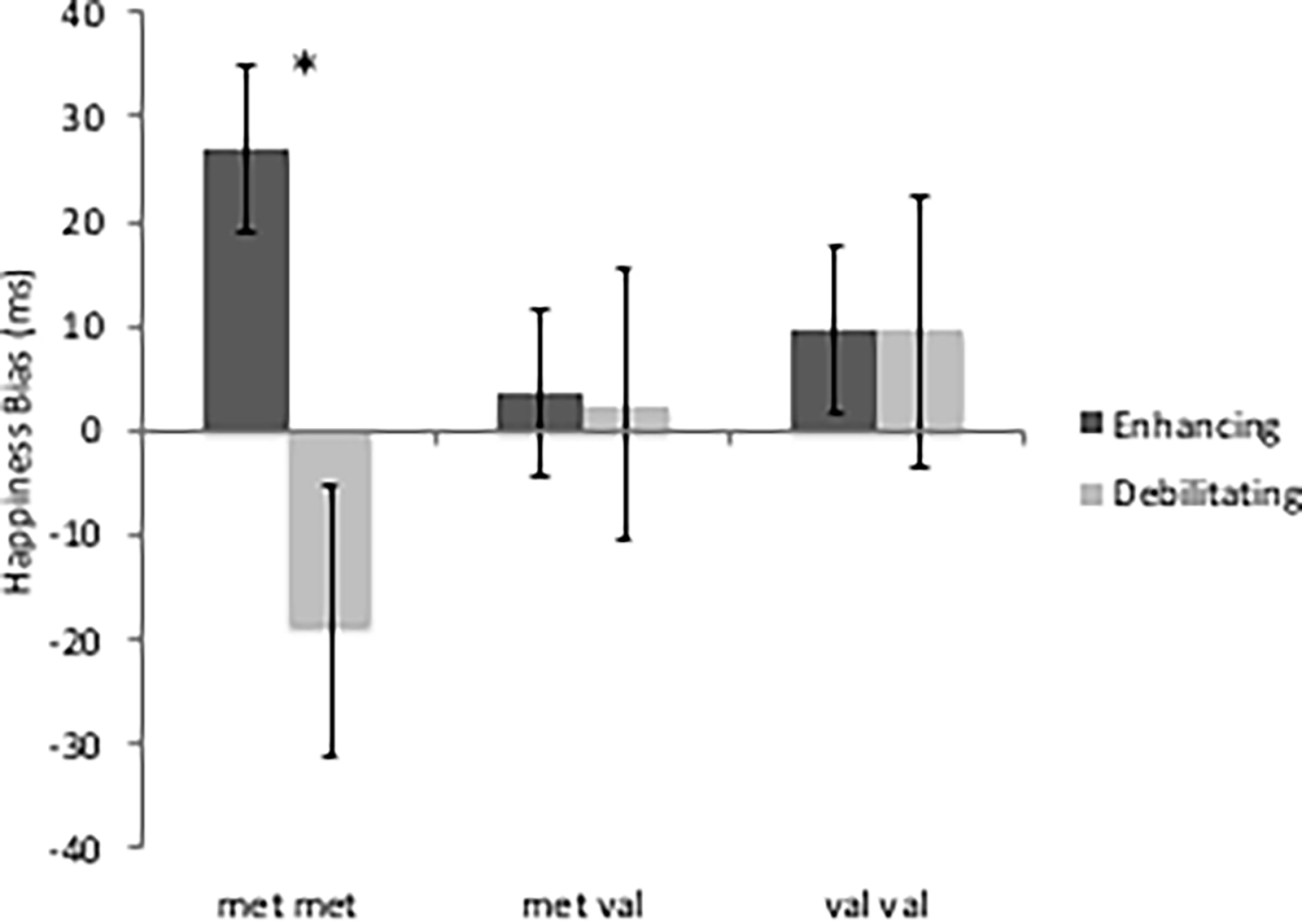
Effect of genotype and mindset condition on happiness bias. The mindset x genotype effect was marginally significant at p **≤** .10. Asterisks indicate the significant difference of the effect of mindset manipulation for each genotype (** *p* **≤**.01; * *p* **≤**.05) revealing that the effect of mindset condition was significant for met/met individuals but not val/val or met/val individuals. Error bars represent standard errors of the means.

We examined the effect of stress mindset and genotype on participants’ attentional bias to happy and angry faces and cognitive interference by conducting a series of univariate ANOVAs. Results for the attentional bias for happy faces yielded a marginally significant mindset x genotype effect (*F*_2,91_ = 2.56, *p* = .084, η^2^ = .061) (Fig 2). Simple effects tests splitting the sample by genotype indicated that the mindset manipulation had a significant effect on happiness bias for met/met individuals (*F*_1,13_ = 7.22, *p* = .028, η^2^ = .474) in that met/met individuals in the SIE condition had more bias towards happy faces and met/met individuals in the SID condition had more bias towards angry faces (Fig 2). Happiness bias did not significantly differ as a function of mindset condition for met/val and val/val participants. Simple effects splitting the sample by mindset condition revealed no significant effects of genotype in either the SID or SIE condition. There was no interaction between mindset and genotype for attentional bias for threat faces.

**Fig 3:**
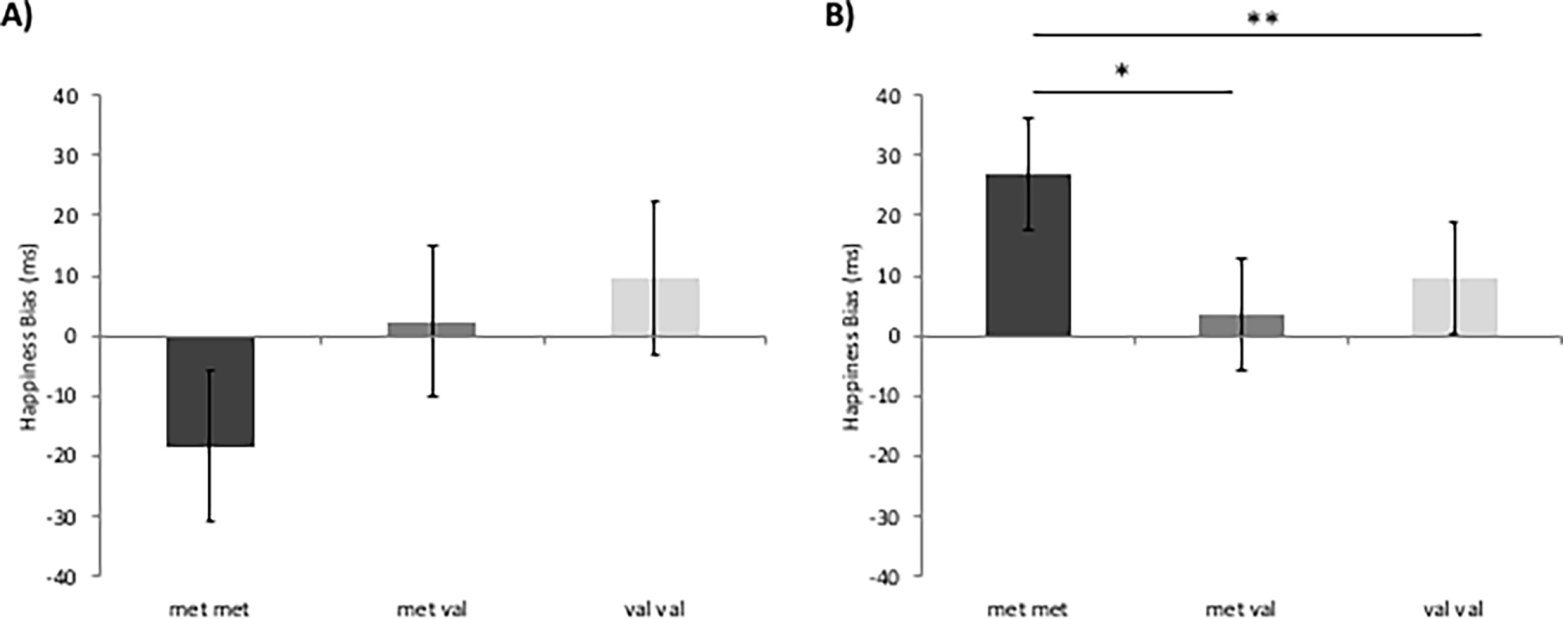
**Effects of genotype on cognitive interference in SIE (A) and SID (B) conditions.** There is a significant genotype effect in the SID condition (p **≤** .01) (B) and not in the SIE condition (A) Asterisks indicate significant differences between genotype in both SIE and SID conditions using Bonferroni corrected post hoc comparisons (** *p* **≤** .01; * *p* **≤** .05) revealing that in the SID condition, met-met individuals experience a cognitive deficit (more interference) compared to both met/val and val/val individuals whereas this deficit is removed in the SIE condition. The time x mindset x genotype effect is significant at p **≤** .05. Error bars represent standard errors of the means.

With respect to cognitive interference (Stroop task), the mindset x genotype effect was significant *F*_2,91_ = 3.09, *p* = .050, η^2^ = .063. Simple effects tests splitting the sample by genotype indicated that the mindset manipulation had a marginally significant effect on cognitive interference for met/met individuals (*F*_1,12_ = 4.46, *p* = .073, η^2^ = .389) whereas there were no significant effects for met/val and val/val individuals. Simple effects tests splitting the sample by mindset condition indicated that there was a significant effect of genotype in the SID condition (*F*_1,51_ = 5.34, *p* = .008, η^2^ = .199) (Fig 3B) but not in the SIE condition (Fig 3A). Bonferonni corrected comparisons revealed that met/met individuals had significantly more cognitive interference than both met/val (p = .049) and val/val (p = .006) (Fig 3B) individuals in the SID condition but that this cognitive deficit was removed and not significant in the SIE condition (Fig 3A). Cognitive interference did not significantly differ as a function of mindset condition for met/val and val/val participants.

## Discussion

Stress has both enhancing and debilitating effects depending on the lens through which it is perceived. Here we present novel evidence suggesting that genetic variation in *COMT* can further modify responses to a stress mindset manipulation. Priming individuals with an SIE mindset had the greatest effects on met/met compared to met/val and val/val individuals. SIE mindset effects on met/met individuals tended to be favorable. This difference between SIE and SID effects by genotype was evident in the significant increases in positive affect and attention, improved cognitive functioning, and bias toward happy faces post-speech found in the met/met SIE group. In comparison the responses of participants with at least one val-allele were not as affected by the mindset manipulation. Taken together, these results align with existing research suggesting that variation in *COMT* modifies the link between social and interpersonal environmental cues and cognitive, emotional, and physiological responses as seen in differential susceptibility [19, 33], suggestibility [34], and response to placebo treatment [20]. Further, our findings suggest that met/met individuals are also more responsive to a stress mindset manipulation. These findings are also consistent with evidence that met/met individuals, compared to val/val individuals, are more susceptible to confirmation bias and are likely to be influenced by and have confidence in explicit initial information/instructions [35].

Stress Mindset Theory holds that individuals who believe stress has enhancing properties are more likely to adaptively engage with stress they are experiencing and therefore experience potential positive benefits such as improvements in performance, health and wellbeing [1]. The results presented here suggest that this pattern of responses was especially true for met/met individuals who exhibited higher positive affect during and after enduring stress whereas val/val and met/val individuals did not.

Previous research has indicated that while met-homozygotes typically outperform val-homozygotes on cognitive tasks in low-stress conditions, met-homozygotes indicate cognitive deficits in high-stress conditions [36–39].This difference is attributed to inhibition of normal cognitive processing by the flood of dopamine into the PFC released during stress [40]. This outcome is particularly the case for met-homozygotes under stress, who experience a further rush of dopamine on top of elevated basal dopamine levels relative to val-homozygotes. In the current study, we found that the stress mindset manipulation effectively mitigated met/met deficits in cognitive functioning under stress, such that whereas met/met individuals in the SID condition exhibited cognitive deficits marked by greater interference on the Stroop task, these deficits were attenuated for met/met individuals in the SIE mindset manipulation in which met/met cognitive interference was similar to that of met/val and val/val individuals. This elimination of a cognitive deficit for met/met individuals was marked by three-fold improvements in cognitive function for met/met individuals in the SIE condition compared to those in the SID condition. These findings align with research showing that met/met individuals display inferior cognitive functioning under stress that results from the overabundance of prefrontal dopamine [37, 41, 42] and suggest that a SIE mindset manipulation may be especially effective in these individuals to boost their cognitive performance and eliminate deficits in cognitive functioning under stress.

Met/met participants in the SIE condition displayed increases in visual attention to positive stimuli; a complete reversal from the met/met participants in the SID condition who showed a bias away from positive faces. Conversely, met/val and val/val participants bias to positive faces was unchanged regardless of the mindset manipulation. These results further support that the effects of stress mindset manipulations on cognitive and affective responses to stress are most pronounced in met/met individuals, however they should be considered with caution as the omnibus mindset x genotype effect was marginal at p **≤** .10 and not the p **≤** .05 level. Hence, these findings suggest that a SIE mindset manipulation may be especially effective in these individuals to boost their cognitive performance and eliminate deficits in cognitive functioning under stress.

In this study, we explore the impact of *COMT* genetic variation on mindset and reaction to stress. Changes in mindset across all participants were concordant with their respective mindset manipulation and did not differ by *COMT* genotype. However, cognitive and affective reactions to stress did differ by genotype, with significant changes among met/met but not val allele carriers. This finding likely derives from the higher levels of prefrontal cortex (PFC) dopamine [43] and circulating stress hormones seen in individuals homozygous for the *COMT* low-activity met allele. Further, compared to val allele carriers, met/met individuals tend to experience higher levels of psychosocial stress [13, 14], be more susceptible to placebos [20] and perform poorly on cognitive tests during high-stress conditions [44, 45]. Hence, our finding that SID condition elicited poor cognitive performance and negative affective response in met/met participants is consistent with parallel behavioral evidence. In contrast, our finding that the SIE intervention was effective at shifting the stress response in met/met individuals from negative to positive is novel. The prefrontal cortex (PFC) is considered the brain region where suggestions and instructions are processed to mediate changes in top-down control and affective meaning [46]. Hence the higher levels of PFC dopamine associated with met/met individuals might have allowed the SIE suggestions and instructions to override prior negative experiences of stress, resulting in responses more in line with a SIE mindset. Although the neurological underpinnings remain to be determined, these findings suggest that neurogenetics coupled with mindset studies might yield further insights into the neurological factors influencing responsiveness to mindset manipulations and the conditions in which they are most likely to be beneficial.

There are several limitations of the present study. Because *COMT* genotype was analyzed after the conclusion of the study, genotypes were not randomly assigned by condition. Reassuringly however, there were no significant differences by genotype across conditions despite the relatively low number of met/met participants. Another possible limitation is that the study was advertised as a “stress and performance” study, which may have been a disincentive for individuals who tend to be more negatively affected by stress and could have resulted in a self-selection bias toward more functional participants. It is therefore possible that participants were a more resilient group of met/met individuals than is represented in the general population. It is also worth noting that the majority (65%) of our sample was women. Sexual dimorphism in COMT resultant from estrogen mediated differences in transcriptional regulation has been implicated in differential genetic associations in behavioral and psychiatric phenotypes among men and women [47]. Hence although we found no effects of sex in this study except for happiness bias (in which case it was controlled for), future research would benefit from using larger populations with more equivalent numbers of men and women such that sex effects can be more adequately explored.

Critically, behavioral phenotypes arise from a complex interplay of multiple genes. Although we are limited here by the examination of a single gene and polymorphism, the functional effects of the rs4680 polymorphism in the dopamine signaling pathway and the abundance of behaviors including placebo response that it modifies make it a model genetic variant with which to launch the exploration of genetic effects on mindset. Future work should aim to better understand other genetic moderators of stress that may be susceptible to manipulations of expectations, such as variation in other genes involved in serotonin signaling and dopamine pathways [48], and using Genome Wide Association Studies (GWAS) to better understand gene by environment interactions for polymorphisms that moderate the availability of key neurotransmitters for affective functioning, behavior, and physiology [49, 50].

Taken together, the results herein add to our understanding of the effects of stress mindset manipulations by suggesting that some of the variability in mindset manipulation effects may be at least in part explained by genetic variation at polymorphism such as *COMT* rs4680. Future work is needed to determine the generalizability of these findings to mindset manipulations outside the domain of stress, such as mindsets about the nature of intelligence as fixed or malleable [51], mindsets about healthy eating as indulgent or depriving [52, 53], or mindsets about willpower as limited or nonlimited [54]. To conclude, we find it critically important to point out that the existence of genetic moderators of mindset effects is not an indicator that these differences are static and uncontrollable. Rather, these differences hint at potential mechanisms linking mindset interventions with outcomes and, as such, can provide important insight for understanding how mindset interventions can be changed to maximize effects where desired (i.e. positive mindset effects) and minimize effects where undesired (i.e. negative mindset effects), regardless of one’s genotype. Thus, although much remains to be explored, these results lay the preliminary groundwork for understanding not only for whom mindset effects are most effective, but why and how these effects may be optimized to improve important physiological, cognitive and affective outcomes.
