# Correction: Catechol-O-Methyltransferase moderates effect of stress mindset on affect and cognition

**DOI:** 10.1371/journal.pone.0216305

**Published:** 2019-05-02

**Authors:** Alia J. Crum, Modupe Akinola, Bradley P. Turnwald, Ted J. Kaptchuk, Kathryn T. Hall

Fig 3 is incorrect. The authors have provided a corrected version here.

**Fig 3:**
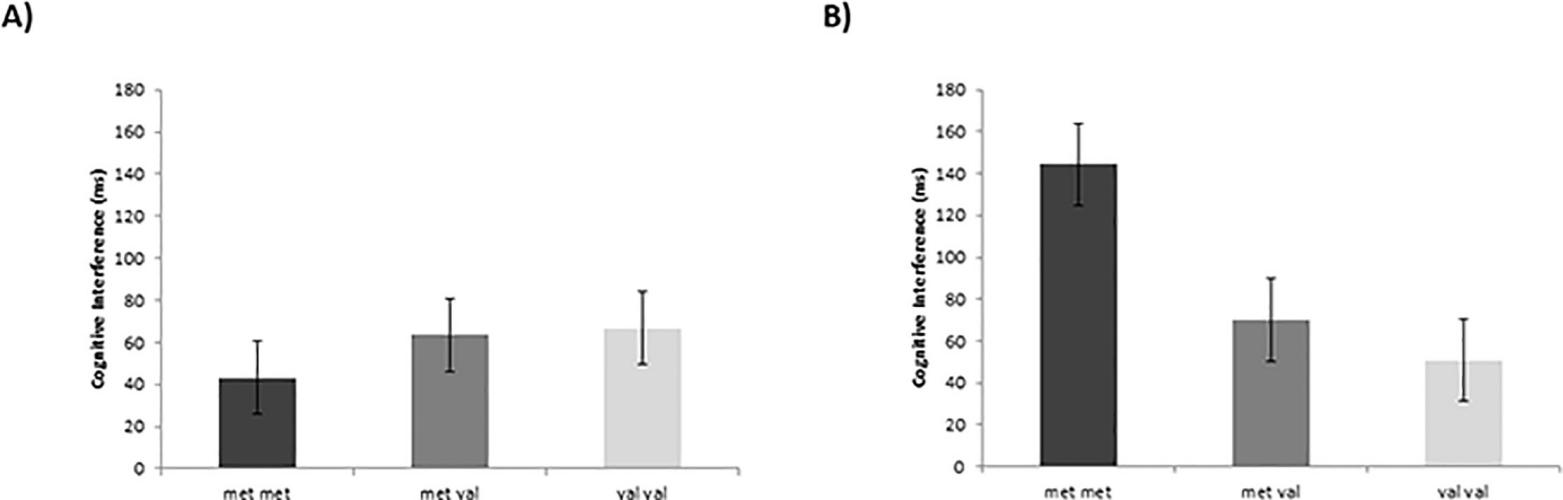
**Effects of genotype on cognitive interference in SIE (A) and SID (B) conditions.** There is a significant genotype effect in the SID condition (*p* **≤** .01) (B) and not in the SIE condition (A) Asterisks indicate significant differences between genotype in both SIE and SID conditions using Bonferroni corrected post hoc comparisons (** *p* **≤** .01; * *p* **≤** .05) revealing that in the SID condition, met-met individuals experience a cognitive deficit (more interference) compared to both met/val and val/val individuals whereas this deficit is removed in the SIE condition. The time x mindset x genotype effect is significant at *p* **≤** .05. Error bars represent standard errors of the means.
